# “Nothing is ever going to change if we don't start advocating for our child.”: Community-level disclosure and stigma management strategies among parents of internationally adopted children living with PHIV

**DOI:** 10.3389/fpubh.2023.1091335

**Published:** 2023-03-17

**Authors:** Amanda Bingaman, Alison Hamilton, Bethany Houpt, Rosemary Olivero, Cynthia Fair

**Affiliations:** ^1^Public Health Studies, Elon University, Elon, NC, United States; ^2^Department of Psychiatry and Biobehavioral Sciences, David Geffen School of Medicine, University of California, Los Angeles, Los Angeles, CA, United States; ^3^Center for the Study of Healthcare Innovation, Implementation and Policy, VA Greater Los Angeles Healthcare System, Los Angeles, CA, United States; ^4^Department of Pediatric Infectious Disease, Helen Devos Children's Hospital, Grand Rapids, MI, United States

**Keywords:** international adoption, perinatally-acquired HIV, community stigma, adoptive parents, adoptee

## Abstract

**Background:**

The number of internationally adopted children living with perinatally-acquired HIV (IACP) in the U.S. is increasing, yet little is known about their families' experiences navigating HIV disclosure within a community context. This paper examines the lived experiences of adoptive parents as they navigate HIV disclosure and manage stigma toward their adopted children within their broader communities.

**Methods:**

A purposive sample of parents of IACP was recruited at two pediatric infectious disease clinics and via closed Facebook groups. Parents completed two semi-structured interviews approximately one year apart. Interview questions included strategies parents used to reduce the impact of community level stigma that their child is likely to encounter as they mature. Interviews were analyzed using Sort and Sift, Think and Shift analytic approach. All parents (n = 24) identified as white and most (*n* = 17) had interracial families, with children adopted from 11 different countries (range: age at adoption 1-15 years; range: age at first interview 2-19 years).

**Results:**

Analyses revealed that parents serve as advocates for their child by both supporting more public HIV disclosure at times, but also applying indirect strategies such as working to improve outdated sex education material. Knowledge of HIV disclosure laws empowered parents to make informed decisions about who, if anyone, in the community needed to know their child's HIV status.

**Conclusion:**

Families with IACP would benefit from HIV disclosure support/training and community-based HIV stigma reduction interventions.

## 1. Introduction

Loosened restrictions on the immigration of individuals living with HIV have contributed to a growing number of internationally adopted children with perinatally-acquired HIV (IACP) in the United States since 2010. Despite such increases, there is an apparent lack of systemic data collection on the population of IACP. Additionally, little is known about the families in the United States who seek to adopt children with perinatally-acquired HIV (PHIV) from other countries. IACP face health needs that are distinct from HIV-negative adoptees (1), and they likely face societal and community stigma around their HIV status.

Along with managing their child's medical care, parents need to make decisions about when and how to disclose their child's HIV status to the child, family, and broader community contexts. Fear of HIV stigma often shapes disclosure decisions (2). Stigma is associated with a variety of adverse outcomes among people living with HIV, including social isolation (3), depression (4–7), and poor quality of life (8). Providers generally suggest prospective adoptive parents prepare for HIV-related stigma from both extended family members and the community (9) and there is extensive research on internalized HIV stigma at the child and family level [e.g., (10, 11)]. However, there is a lack of understanding and research focused on community-level HIV stigma and the lived experiences of adoptive parents in the United States as they navigate HIV disclosure and manage stigma at the community level. Research by Turan et al. (12) underscores the importance of focusing on community level HIV stigma as those who experience stigma in their community are more likely to internalize stigma leading to adverse outcomes.

Community-level HIV disclosure refers to groups or individuals outside the nuclear family such as school, church, or other community organizations. Consequences of community-level disclosure include public ridicule, rejection, and breaches of confidentiality (13). On the other hand, maintaining secrecy of one's HIV diagnosis can lead to guilt, shame, and anxiety. Logie et al. (14) argues that a dual focus on the consequences of HIV stigma as well as how communities address stigma allows for a more complex understanding of stigma. Previous research has examined the strategies used to manage stigma and disclosure to the child and family (2). Findings revealed that anticipated and experienced stigma shaped disclosure practices. Yet no research has examined the ways in which adoptive parents manage stigma and disclosure practices to the broader community. Addressing HIV stigma at a community level may be difficult (15). Belden et al. (16) found that even community-based organizations designed to support those living with HIV have few strategies to combat stigma.

This study aims to address how adoptive parents of IACP prepare their children for potential stigma from the community. More specifically, it examined the decisions parents make regarding disclosure of HIV to the community and analyzes the strategies parents use to mitigate the effects of HIV-related stigma on their adopted child.

## 2. Methods

### 2.1. Participants

Parents were eligible if they had an IACP of any age in their care for at least one year. Participants were recruited from the Helen DeVos Children's Hospital, Seattle Children's Hospital, and closed Facebook groups through a purposive snowball sample. Flyers explaining the research study were distributed to eligible parents in the clinics and those interested contacted researchers. Each parent was asked if they knew of another adoptive family who would be willing to participate. Several clinic parents shared flyers with other families via social media. Those interested in participating reached out to the researchers. All parents were aware of their adopted child's HIV status and intentionally sought to adopt a foreign-born child with HIV.

Twenty-eight parents expressed interest in participating. Twenty-four parents representing 23 family units living in the United States completed the interviews. Four parents who originally wished to participate did not respond to the follow up email designed to set up the first interview. All parents participated in both sets of interviews, one year apart. Twenty-three of the 24 participating parents were mothers. Fathers were typically not available during the interview and mothers were primarily responsible for child health management tasks. Seventeen families also had biological children both older and younger than their adopted sibling. Three of the 23 family units were interracial families where the parents identified as white and the children were a different race. See Table 1 for additional demographic information.

**Table 1:** Parent characteristics.

**Demographic variable (*n* = 24)**	***n* (%)**
**Race/ethnicity**	
White	24 (100)
**Sex**	
Female	23 (96)
**Marital status**	
Married	23 (96)
**Religion**	
Christian	23 (96)
None/atheist	1 (4)
**U.S. Geographic region of home**	
Midwest	11
West	8
Southeast	3
Northeast	2
**Age**	Mean 37.9 years (range 29-54)
**Biological children in the family**	
Yes	17 (71)
	Mean 2.6 children (range 0-5)
**Number of adopted children/family**	Mean 2.2 children (range 1–7)

IACP originated from 10 different countries, the most common of which was Ethiopia. Median age at adoption was 7 years (range 1–15). See Table 2 for additional information.

**Table 2:** Adoptee characteristics.

**Demographic variable (*n* = 27)**	***n* (%)**
**Sex**	
Female	13 (48)
Male	14 (52)
**Adoptee country of origin**	Number of children from each country
Ethiopia	9 (33)
Russia	4 (15)
Uganda	4 (15)
Ukraine	2 (7)
India	2 (7)
China	1 (4)
Columbia	1 (4)
Georgia	1 (4)
Haiti	1 (4)
South Africa	1 (4)
Zambia	1 (4)
Age at first interview	Median 11 years (range 2–19)
Age at adoption	Median 7 years (1–15)

### 2.2. Procedure

This qualitative study used in-depth interview methods to collect data. Data were derived from two 60 min semi-structured audio-recorded phone interviews. All interviews were held in English and conducted via phone due to the wide geographic distribution of families who came from 13 states. Parents also preferred phone interview to preserve confidentiality. Interviews were facilitated by four trained interviewers based at Elon University. Authors AB and CF conducted the interviews along with one undergraduate and one graduate student. The first round of interviews focused on the adoption story and HIV disclosure decisions, and the second round explored child adjustment and changes to adoption and HIV disclosure narratives. Interviewers used a semi-structured interview schedule that included three primary sections: demographic information including child's medical history, process of adoption, and HIV disclosure experiences including disclosure to child, immediate family, and community context such as school and church.

This study was approved by the Elon University's Institutional Review Board. Participants gave oral informed consent prior to each interview and received a $25 gift card as a thank you for their time.

### 2.3. Analysis

Interviews were recorded, transcribed and analyzed using the Sort and Sift, Think and Shift analytic approach (17). First, transcripts were read thoroughly by AB and CF, who participated in a week-long qualitative analysis workshop, to identify preliminary themes related to family communication and disclosure (18). Structured episode profiles were then developed by summarizing each transcript in a template (17). Episode profiles were transferred to matrices to identify places of convergence and divergence across interviews (19). Upon completion of the matrix analysis, the transcripts were uploaded into Dedoose, a qualitative software package (20). Codes and sub-codes were developed from matrix analysis and consensus-based coding was conducted by [AB and CF]. None of the participants' names were associated with responses. Pseudonyms were assigned when necessary.

## 3. Results

Disclosure of HIV to the child, nuclear and extended family members, and broader community was one of the most difficult challenges faced by families of IACP. In response, parents developed strategies and practices to manage HIV stigma, which varied based upon whether they sought to address stigma with their child vs. with family or those outside the home.

## 4. Community level stigma management strategies

Beyond child and family factors, parents considered the broader legal and social factors that influenced their approaches to stigma. Demographics of communities played a role in whether parents chose to share the HIV status of their child who was often the only child of color in a predominantly white neighborhood. School was the most common place to avoid disclosure, as was church. Six families disclosed at settings such as church, while others did not feel comfortable doing so. Parents were aware of whether HIV disclosure was legally mandated in their state.

Parents reflected upon the importance of advocacy and possible broader positive impacts of disclosure. Advocacy most often took the form of trying to share accurate HIV knowledge as a strategy to combat stigma. Many struggled with the decision of whether to disclose their child's status to the community. All parents wanted to advocate for their child to combat HIV stigma, but were hesitant to reveal their child's status, expressing, “I feel like I can't”. One parent explained that the nuclear family “really went back and forth that we're going to make her our poster child, to be an advocate and un-stigmatize this whole thing, or let it be her story to share”. In contrast, another parent confidently stated, “I will have no problem sharing if she [child] gives me that permission”, suggesting that some parents hoped their children would want to become advocates as they grew older. The decision of whether to disclose to the community appeared as a “double standard” for the parents and parents expressed an “internal struggle” when disclosing to community members: “…say, ‘Oh yes, this is not a big deal, but don't tell anybody”. Regardless of the level of openness a family chose, all parents acknowledged the harmful reality of HIV stigma, its impact on their child, and how they chose to approach disclosure. Most parents took their cues regarding public disclosure from their child noting it is “ultimately their decision because it's their medical history”.

“*Nothing is ever going to change if we don't start advocating for our child”*. Some parents combatted HIV stigma by advocating publicly for their child, while others attempted to change HIV school curriculums that perpetuated outdated information to reduce levels of community stigma should their child wish to disclose their HIV status in school. One parent shared they had been working “in the background... to pull the video that they normally show [in Sex Ed] and substitute it”. Addressing HIV stigma and other forms of racial discrimination often coincided with the desire to protect their child, as one parent recounted: “Mama bear comes out and I fight school wise. But at the same time, some of them don't want me fighting”. This experience demonstrates the balance some parents struggled to find when determining how and when to intervene in discriminatory situations.

Some parents chose to openly disclose to schools, churches, etc. because they “feel it's our responsibility to normalize it for them, rather than allowing the stigma to continue until he's an adult”. These parents typically used disclosure to educate others and combat HIV stigma, an act of advocacy they hoped their younger children would join them in once they were adolescents or young adults. One parent expressed, “My hope is that she will be her own advocate and that she will want to speak out against the stigma and we will 100% jump onboard with that when she is ready”.

One parent “shared factual information as much as possible and also just let people see our comfort level with the diagnosis”, noting “that's had the biggest impact”. Another parent explained, “we don't advocate for total non-disclosure because... if it's seen as a taboo thing by even us, then there's really no hope for the general population”. It was important to some parents that their children were able to “impact their friends”. One mother outlined her responsibility to advocate for her child:

I need to make this world a better place for my kids. That's why I'm her mom, right? I just don't ever think that anything is by coincidence, and I know that she's in our family because the Lord graciously placed her with us, and He knew I'd be a fighter for her.

“*Legally there's a lot of stuff you don't have to disclose”*. Existing policies and laws were also a tool that parents utilized to combat stigma. Parents stressed the importance of “knowing your rights” as a measure for maintaining their child's privacy, especially with disclosing information on medical forms for school, church, and sports teams. Some parents voiced that not all medical professionals realize “by law, we do not have to tell anybody”. Most parents advocated for universal precautions, emphasizing that their child should not be treated differently and that there would be no need to disclose if they were practiced. One parent reasoned, “He's undetectable, they're supposed to use universal precautions. So, to this point I have not disclosed with school”. Similarly, another parent stated, “If everyone does universal then we don't have to tell anyone”.

“*It's hard enough to be Black here”*. Parents managed additional stigma stemming from environmental factors such as the size of the community, degree of diversity, and political leaning with varying levels of comfort. The stigma management strategy of maintaining privacy was highly shaped by the fear of unintentional exposure and compounding stigma of racism. One family shared, “We disclosed more than I would have looking back. And now we live somewhere different, and my son absolutely does not want anybody to know”, highlighting the impact of the environment and community on the extent to which a family may disclose their child's status to others. Parents acknowledged concerns on maintaining privacy of the information due to the town's small size. For example, one family described that they live in a “pretty small, rural area, and we don't disclose to very many people”. One common specific stigma management strategy included arranging for their child's medication to be delivered to the house instead of going in person to pick it up from the local pharmacy to avoid disclosure.

Some parents argued that their child already faced other types of discrimination in the community due to their race or adoption status. White parents do not have direct experience with racism. Those with inter-racial families noted that they had to learn how to talk with their child of color about racism and how to combat racist acts or comments. Parents understood that information may travel fast through a community, and one mother expressed she did not want to contribute more information such as HIV status that would single out her child because “it's already gone around at school that our children are adopted”. Some parents reported that their children experienced racist comments on school buses, classrooms, or in the community, and did not want to “throw one more label on her.” Parents noted that their children focused on trying to find their place in their adoptive family and school environments, and that they “had enough to deal with in that”.

“*The benefits, obviously, are feeling connected and supported, having a community”*. By contrast, some families managed stigma by disclosing HIV to others, explaining they drew comfort in the fact that others knew their child's status. For example, one parent explained, “I just never wanted anyone to come in contact with being in her care and not know”. Families created support systems for themselves in times of need. One parent shared,

We were so depleted already and so far out of our comfort zone and just so in need of people to support us and encourage us when we came home, we just were willing to open up I think a little bit more and share.

This family's experience emphasizes the need for support during the time of an adoption, and how disclosure can construct that support. Another parent shared, “When it first happened, I disclosed more, but now I don't even need to because it's not something that really weighs on me”, illustrating how disclosure practices and stigma management methods can change over time depending on a family's needs. Another parent explained that over time she felt “more just confident in where we are in that situation in disclosure and less like I have to hide it. But also, I don't feel comfortable just making it... telling the worl”.

## 5. Discussion

The purpose of this study was to analyze decisions adoptive parents make regarding disclosure of HIV to their communities and examine the strategies parents used to mitigate the effects of HIV-related stigma on their child. Parents expressed fear of stigma, particularly the layered nature of stigma and were active participants in trying to mitigate against negative consequences for their child. Advocacy played a critical role in preparing for the more public disclosure of the child's HIV status with their child's permission. Parents were aware that they may not be able to control when/if their child's HIV status was disclosed and balanced advocating for their child while maintaining confidentiality. This struggle suggests the need for targeted community-based interventions designed to support IACP and their families.

Most parents stressed that disclosure to others was their child's decision. However, some were more vocal about being their child's advocate and actively sought to address HIV-related stigma through school or community-based education. Some took a more indirect approach working with local schools to revise outdated sex education, which typically portrayed HIV as a “death sentence”, without disclosing their child's HIV status. They also hoped their child would join them in actively fighting against HIV stigma in what they believed would be an empowering experience for their child. Parents felt empowered by understanding disclosure laws in their state so they could make informed decisions about disclosure outside the family. Similarly, Fair and Ginsburg (21) found that individuals living with HIV who had high knowledge of legal rights scored significantly lower on disclosure concerns.

Both parents and health care providers should be aware of the demographic characteristics of the outside community and how they influence disclosure decisions as well as contribute to potential stigma from the environment. Such awareness can lead to productive conversations about when and if to disclose more publicly. Families who lived in more rural and homogeneous communities frequently expressed concern over the potential breach of confidentiality at local pharmacies. Prior research indicates that negative experiences related to HIV stigma, especially in health care settings (22), are not limited to the families in the present study, indicating a widespread need for stigma reduction.

White adoptive parents have not personally experienced racism and acknowledged this was an added challenge in parenting within an inter-racial family. As a result, families may need support from others to help their child navigate experiences of racism. HIV stigma is inextricably linked with other stigmas such as racism, and white adoptive parents sought to help their child navigate predominantly white spaces which led several parents to not disclose their child's HIV status as it would simply add another layer of stigma. Indeed, several parents in the current study did not want to disclose their child's status because their child already experienced racial discrimination and they did not want to add another source of discrimination. This concern is supported by documented experiences of intersectional stigma, which involves compounding social prejudices such as homophobia in addition to HIV stigma (23) as well as research on healthcare providers' views of challenges experienced by parents who have IACP (9).

On the other hand, several families described positive experiences when disclosing to community members. Such disclosure can reduce the shame experienced by maintaining the secret of one's HIV status (24). Families who disclosed more publicly felt their disclosure was a way to combat HIV stigma and improve HIV education. This aligns with Wiener et al.'s (25) research with families who made the decision to “go public” with their child's HIV status through news or other media outlets. However, results revealed that some parents who had shared their child's HIV status more publicly regretted that decision, especially as their child matured and expressed a desire to remain private about their diagnosis.

Findings from the current study have implications for adoptive parents and health care providers. For example, parents may require support from providers and other professionals on how to best support their child. Infectious disease care providers can assist with ongoing education should the family or child decide to share the diagnosis publicly. They can also serve as a resource for parents who seek to increase levels of accurate HIV knowledge without disclosing their child's HIV status. Further, they can serve as a sounding board as parents wrestle with the pros and cons of community disclosure. Addressing community level stigma is critical as Turan et al. (12) outlined the cyclical relationship between community stigma, which led to internalized stigma and ultimately resulted in worse outcomes such as lower adherence to antiretroviral treatment and self-esteem as well as worse social support among individuals living with HIV.

Findings of this study should be considered in light of several limitations. The significant geographic variability in the study sample makes it difficult to apply to other communities. Further, only parent voices were included. Lastly, all parents in this study identified as white, and most as Christian, limiting the diversity of our sample. It is unclear if more diverse families are also caring for IACP. The high proportion of interracial families in this study is consistent with international adoption in the United States. In 2007, the Office of the Assistant Secretary for Planning and Evaluation reported that 84% of international adoptions in the United States were interracial adoptions (26). The wide age range of adopted children highlighted how experiences with community-based strategies to mitigate HIV stigma may change over time due to the child's developmental stage.

Despite limitations, this research offers rich insight into the experiences of families who have adopted a child with PHIV from outside the U.S., while most existing studies on IACP focus on the medical and psychological outcomes (1). The current study illustrates the measures parents took to empower and protect their child and deserve careful consideration as they have the potential to impact their child's own management of HIV stigma. Parents served as advocates for their child and worked to create more inclusive environments in healthcare, education, and community settings. Parents may need support as they navigate the balance between advocacy and privacy.

Future research should focus on community-level stigma reduction efforts that reach many different stakeholders. Interventions should be tailored to the age of the child or to where a family is in their adoption journey due to our findings that disclosure and needs of the family change over time. Further, it will be important to amplify the voices of the adoptees as well as the biological children in families to explore sibling dynamics and support. Their reactions to stigma-management strategies would offer valuable information for future interventions.

## Data availability statement

The raw data supporting the conclusions of this article will be made available by the authors, without undue reservation.

## Ethics statement

The studies involving human participants were reviewed and approved by Elon University Institutional Review Board. The patients/participants provided their written informed consent to participate in this study.

## Author contributions

CF, AB, and RO conceptualized the study. RO facilitated participant recruitment. CF and AH conducted qualitative interviews. CF supervised qualitative data collection and data management. CF and AB read interview transcripts and developed content categories from initial concepts. AB subsequently developed episode profiles and coded interviews in Dedoose with CF's supervision. AH provided analytical consultation and insight. AB wrote the first draft of results along with CF and AH. BH wrote the introduction and discussion and reviewed results. AB, BH, and RO reviewed drafts and provided substantial edits. All authors contributed to the article and approved the submitted version.
